# Extracting Primary Open-Angle Glaucoma from Electronic Medical Records for Genetic Association Studies

**DOI:** 10.1371/journal.pone.0127817

**Published:** 2015-06-10

**Authors:** Nicole A. Restrepo, Eric Farber-Eger, Robert Goodloe, Jonathan L. Haines, Dana C. Crawford

**Affiliations:** 1 Center for Human Genetics Research, Vanderbilt University, Nashville, Tennessee, United States of America; 2 Department of Epidemiology & Biostatistics, Institute for Computational Biology, Case Western Reserve University, Cleveland, Ohio, United States of America; The University of Tennessee Health Science Center, UNITED STATES

## Abstract

Electronic medical records (EMRs) are being widely implemented for use in genetic and genomic studies. As a phenotypic rich resource, EMRs provide researchers with the opportunity to identify disease cohorts and perform genotype-phenotype association studies. The Epidemiologic Architecture for Genes Linked to Environment (EAGLE) study, as part of the Population Architecture using Genomics and Epidemiology (PAGE) I study, has genotyped more than 15,000 individuals of diverse genetic ancestry in BioVU, the Vanderbilt University Medical Center’s biorepository linked to a de-identified version of the EMR (EAGLE BioVU). Here we develop and deploy an algorithm utilizing data mining techniques to identify primary open-angle glaucoma (POAG) in African Americans from EAGLE BioVU for genetic association studies. The algorithm described here was designed using a combination of diagnostic codes, current procedural terminology billing codes, and free text searches to identify POAG status in situations where gold-standard digital photography cannot be accessed. The case algorithm identified 267 potential POAG subjects but underperformed after manual review with a positive predictive value of 51.6% and an accuracy of 76.3%. The control algorithm identified controls with a negative predictive value of 98.3%. Although the case algorithm requires more downstream manual review for use in large-scale studies, it provides a basis by which to extract a specific clinical subtype of glaucoma from EMRs in the absence of digital photographs.

## Introduction

The use of electronic medical records (EMR) in biomedical research has gained traction in recent years, driven in part by the large-scale collection of biospecimens associated with EMRs. When linked to extensive biobanks, these large, data-dense resources can be utilized in large-scale genetic and genomic studies[1]. Compared to traditional epidemiological cohorts, which are expensive and can take years to decades to collect, EMRs are a relatively cost efficient alternative with years of medical information immediately accessible. These medical records can be interrogated for a range of phenotypes often not available or included in epidemiological cohorts. Also, large sample sizes can be acquired from the development of phenotype algorithms standardized for use across multiple institutions[2,3].

One such EMR resource is the Vanderbilt University Medical Center (VUMC) Synthetic Derivative (SD). The SD is a de-identified version of Vanderbilt’s EMR and contains inpatient and outpatient medical records collected at VUMC and affiliated clinics. Patient records consist of both structured (e.g., billing codes, procedure codes, laboratory values) and unstructured (e.g., clinical free text) data. To date, the Vanderbilt EMR contains over 2.2 million records with each record containing on average 6.5 years of medical history and an average of eight prescriptions. The SD is linked with VUMC’s DNA repository known as BioVU[4]. These DNA samples are extracted from discarded blood samples collected from outpatient clinical laboratories. The SD in conjunction with BioVU has been used recently in several genetic association studies to identify genetic variants associated with a range of phenotypes such as multiple sclerosis[5], type 2 diabetes[6], cancer[7,8], electrocardiographic traits[9–11], pharmacogenomic-related outcomes[12–14], clinical quantitative traits[15–20], and hypothyroidism[21]. As part of the Population Architecture using Genomics and Epidemiology (PAGE) I study[22], we as the Epidemiologic Architecture for Genes Linked to Environment (EAGLE) study accessed ~15,000 DNA samples from non-European descent individuals in BioVU (EAGLE BioVU) to perform genetic association studies in diverse populations[23].

Here we introduce an algorithm to extract primary open-angle glaucoma (POAG) in EAGLE BioVU to identify cases and controls among African Americans (n = 11,521) for genetic association studies. POAG is a clinical subtype of glaucoma, a heterogeneous group of eye diseases characterized by chronic degeneration of the optic nerve and gradual vision-loss. As the second leading cause of blindness in the United States[24], glaucoma is a leading cause of vision disability. The most common form of this disease is POAG which disproportionately afflicts African Americans. The prevalence of POAG in African Americans is nearly double that observed in European-descent populations[25–27] with rates as high as 5.6% vs. 1.7%, respectively, in individuals over the age of 40 years[28].

A major challenge associated with use of the Vanderbilt EMR for POAG research is that the specialty eye clinic (the Vanderbilt Eye Institute or VEI) currently lacks an interface with structured data fields to digitally upload test results and forms into the broader EMR. Due to this lack of structured data fields and difficulty in de-identifying photographs, test results and digital photographs are not readily available for research use in the SD. Given these limitations, we sought to develop a phenotype algorithm using searchable and parsable elements available in the SD. With this goal in mind, we have 1) developed and implemented a data-mining algorithm to classify individuals as POAG cases or controls; and 2) manually verified the case/control status of individuals to evaluate the algorithm’s performance. Overall, the algorithm identified 267 cases and 4813 controls among African Americans from EAGLE BioVU. After manual review we determined that the algorithm only had a positive predictive value of 51.6% to identify definite POAG cases with an accuracy of 76.3%. Controls were identified with a negative predictive value of 98.3%. Despite the limited positive predictive value of the algorithm in defining POAG case status, our strategy overall provides a starting point in the absence of digital photographs (i.e., the gold standard) to identify definitive cases of POAG from amongst over two million clinical records.

## Results

### Algorithm development

**Fig 1:**
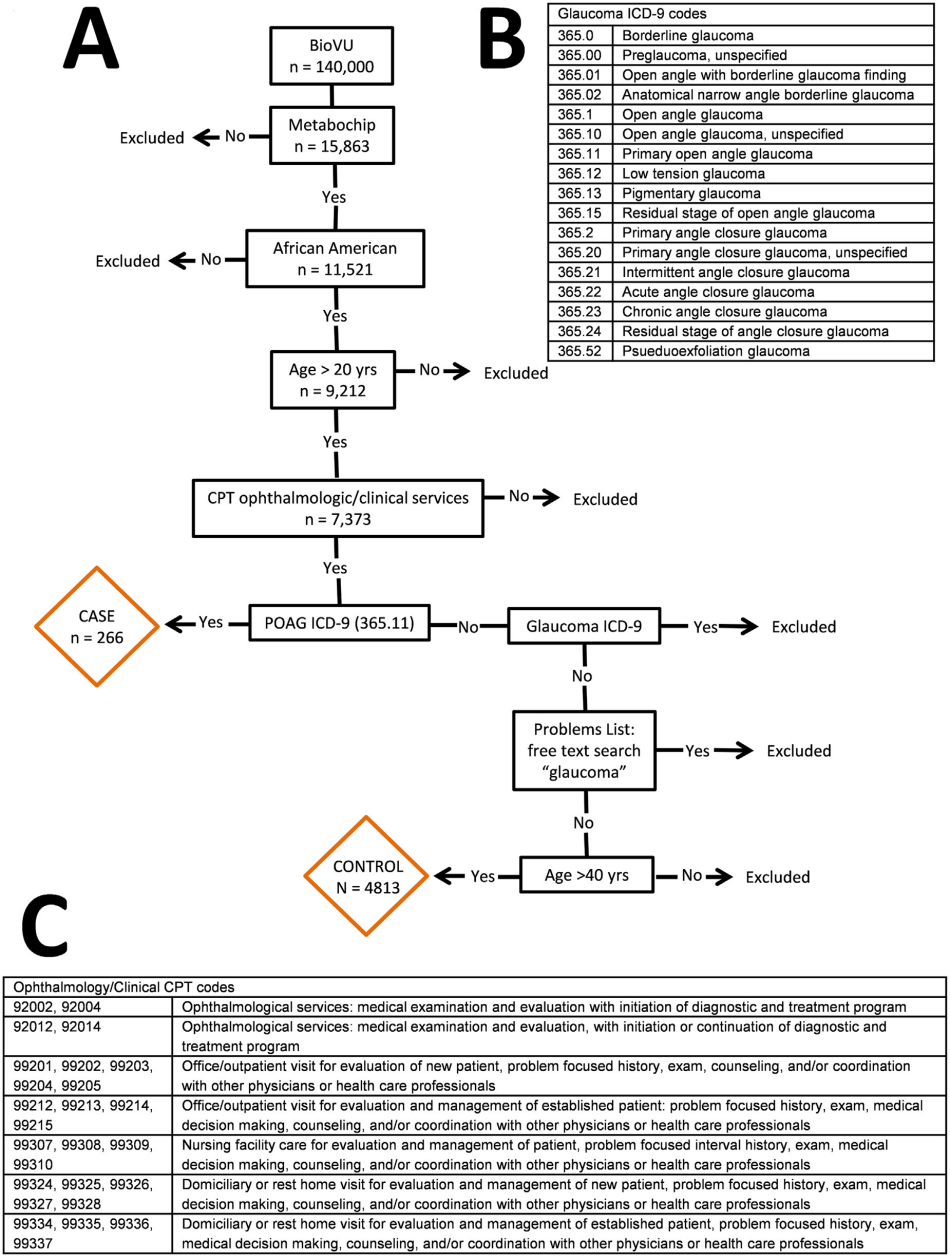
Criteria and decision tree used to classify individuals in EAGLE BioVU as POAG cases or controls. (A) Flow diagram of phenotype algorithm for POAG cases and controls. (B) List of glaucoma ICD-9 codes (C) List of CPT codes for ophthalmology and general clinic procedures.

The full description of the POAG algorithm is available in **Methods**. Briefly, we developed an algorithm designed to identify POAG cases and controls from EAGLE BioVU[23], a subset of records and DNA samples from the larger Vanderbilt SD and BioVU, using a combination of International Classification of Diseases (ICD-9) diagnostic codes for glaucoma, Current Procedural Terminology (CPT) billing codes for ophthalmology/general clinic, and free text searches (Fig 1). Digital photographs taken over the course of multiple visits are the gold standard for diagnosing ocular diseases such as POAG; however these are not readily available in the SD for research as the VEI currently uses a manual/paper process for evaluating and recording the results of a patient’s vision exams. Most of these forms are scanned and uploaded into the EMR as portable document format (PDF) forms. Because it is difficult to de-identify and parse PDF forms, this POAG case/control algorithm targeted structured and easily parsed unstructured data and was designed to maximize the number of individuals eligible for manual review to confirm case/control status.

### Manual Review and algorithm performance

**Fig 2:**
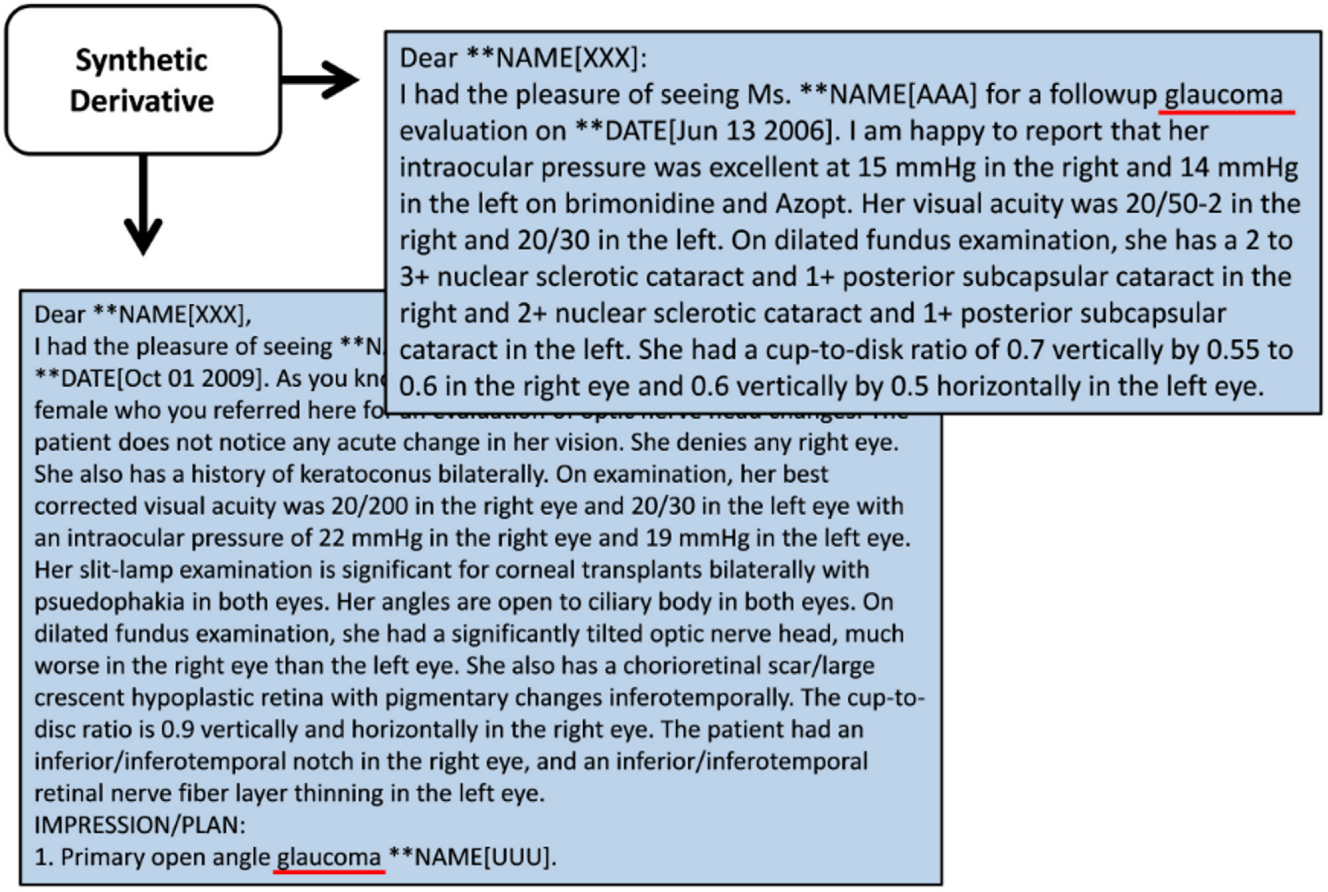
De-identified clinic notes extracted from the Synthetic Derivative. De-identified letters from the Vanderbilt Eye Institute’s ophthalmologists and optometrists extracted from the Synthetic Derivative. These letters represent the primary type of record used to verify POAG case status in EAGLE BioVU. It is an example of a definite case which has the specific clinical sub-type of glaucoma clearly stated and of a potential case which only includes a more general glaucoma diagnosis statement.

The SD records of all EAGLE BioVU POAG cases and a random sample of controls identified by the algorithm were manually reviewed by a single investigator to verify POAG case/control status. Positive predictive value (PPV), negative predictive value (NPV), and accuracy were then calculated to evaluate the algorithm’s performance. Of the 267 individuals identified as cases by the algorithm, 138 were determined to be definite cases based on manual review of records retrieved from the SD. The records accessed for verification of case status were primarily composed of but not limited to surgical reports, optometry, and ophthalmology clinic notes. Other data that were taken into account were medication lists, general and specialty clinic reports, clinical communications, and problems lists. An individual was classified as a definite case if he/she met one of two criteria: 1) a written diagnosis by a Vanderbilt ophthalmologist/optometrist as pertained to a patient’s exact clinical sub-type of glaucoma (An example of this is given in Fig 2) and 2) the patient’s medical record contained *all* of the following: at least two independent mentions of the POAG ICD-9 code (365.11), a glaucoma medication (S1 Table), and a surgical procedure for treatment of POAG complications as identified by a surgical report. Surgical procedures for treatment of POAG may include argon laser trabeculoplasty, selective laser trabeculoplasty, MicroPulse laser trabeculoplasty, and Ex-Press Mini Glaucoma Shunt. Previous studies which successfully developed phenotype algorithms with high PPV for ocular traits found that CPT codes for surgical procedures were sufficient for positively identifying individuals with cataracts[29]. We classified individuals whose records were positive for glaucoma case status but lacked sufficient clinical records to determine the sub-type of glaucoma as “potential cases.” The criteria for potential cases includes *all* of the following: at least one mention of POAG ICD-9 (365.11), an ophthalmology/fundus CPT code (i.e. 92012, 92014, or 92250), glaucoma medication, and a text mention of “POAG” or “glaucoma” in a clinic note or problems history. Of the records reviewed, we identified 67 potential cases. Sixty-two individuals were determined to be false positives. Upon examination of the records, 28 of the 62 false positives were found to be diagnosed with another clinical sub-type of glaucoma, such as uveitic (n = 6), chronic angle closure (n = 11), pediatric (n = 2), neovascular (n = 6), or glaucoma steroid responder (n = 3). Reviews of other false positives lacked sufficient records or communications to determine glaucoma/POAG status. Ambiguous notes included those that state that a patient had “advanced glaucoma,” “ocular hypertension,” or was a “glaucoma suspect.” These individuals were excluded from case status as we could not reasonably verify a diagnosis.

For the POAG case algorithm, we determined that the PPV for definite cases was 51.6% with an accuracy of 76.3% (Table 1). When including potential cases, PPV was 76.7% with an accuracy of 83.1% (Table 1). We manually reviewed the SD medical records of 300 randomly selected controls identified by the algorithm to calculate NPV. Of the 300 individuals identified as controls with this algorithm (Fig 1), five showed evidence of glaucoma at the time of review or else had mention of glaucoma in their records. As an additional review function, we performed a free-text search in all available documents for mention of the following words and abbreviations: glaucoma, fundus, opth, ophth, and vision. The shorthand identifiers such as fundus, opth, ophth [i.e., funduscopic eye exam, ophthalmology clinic, and opth (common abbreviation for ophthalmology)] are used throughout the clinical records especially within communications across clinics. This function was performed to ensure that algorithm-identified controls without qualifying ICD-9 or CPT codes also did not have evidence of POAG in the free clinical text. Performance of the POAG control algorithm was found to have a NPV of 98.3% (Table 1).

**Table 1:** Evaluation of primary open-angle glaucoma phenotype algorithm in African Americans from EAGLE BioVU.

	Sample Size	Manually reviewed	PPV	NPV	Accuracy
Cases	267	267	-	-	-
-Definite		138	51.6%	-	76.3%
-Potential		67	76.7%	-	83.1%
Controls	4813	300	-	98.3%	-

Definite cases were individuals whose POAG status could be determined with high likelihood. Potential cases were individuals whose medical records lacked sufficient information to make a definitive decision. Potential case results were calculated by including both potential and definite case numbers.

### Cup-to-disc ratio (CDR)

As mentioned, ocular phenotypes are difficult to extract from the VUMC EMR due to the lack of structured data fields with the VEI EMR interface. Extracting quantitative phenotypes such as cup-to-disc ratios, the ratio of optic cup diameter to optic disc diameter, proved particularly challenging as there is not a uniform field or manner in which ophthalmologists report these data. We have found, however, that these data can be extracted via expression matching from referral letters and clinic notes sent between ophthalmologists and clinicians in the process of patient care (Fig 2). The following key word phrases were included in the search in reference to the right eye (O.D.) and left eye (O.S.): CDR, cup-to-disc ratio, cup-to-disk, and cup-to-disk ratio. Upon manual review of the results it became apparent that a more sophisticated approach was necessary to differentiate whether the numbers recorded were for the horizontal optic cup ratio, the vertical optic cup ratio, or the quotient of the horizontal-to-vertical CDR which ophthalmologists report interchangeably. When multiple measures of CDR were available for a patient, the most recent measurement was taken.

**Fig 3:**
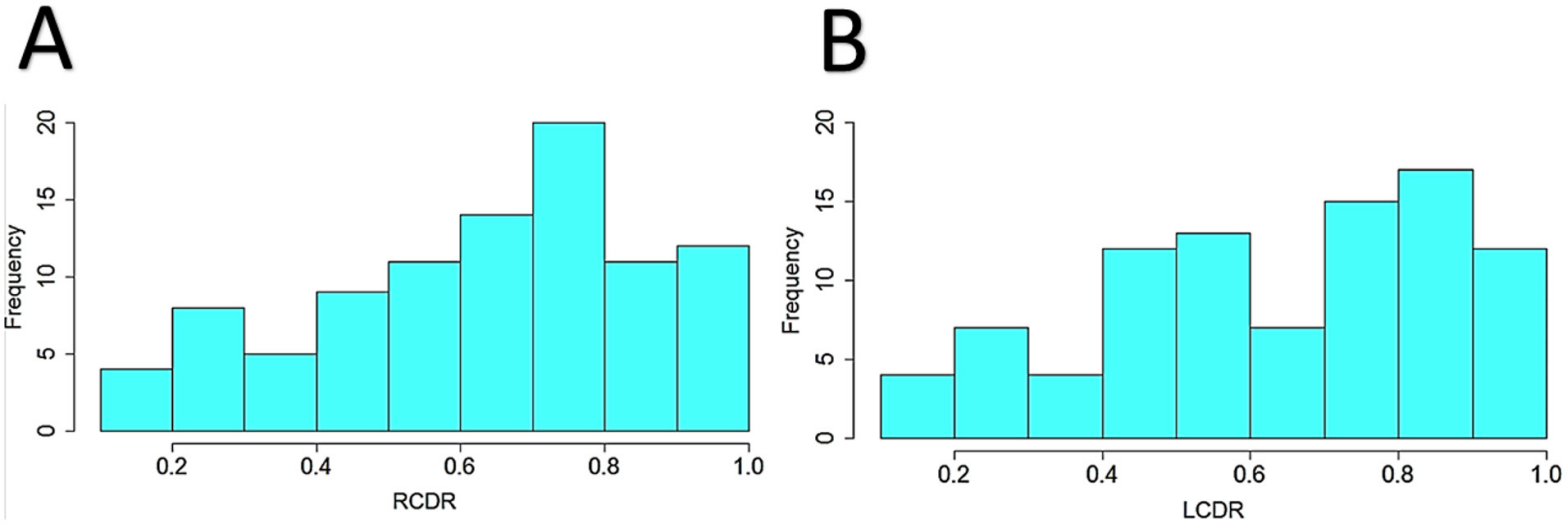
Histogram plots of the distribution of cup-to-disk (CDR) ratios of POAG cases from the Synthetic Derivative. Distribution of CDR for the right eyes (A) and the left eyes (B) of POAG cases from numbers that were manually extracted from the SD.

A total of 132 POAG case records were positive for a CDR key word phrase, and all were manually reviewed for CDR. Only 9 of the 132 records (7%) were missing a measure for CDR. The median values for CDR in this study are 0.7 (SD 0.22) in the right eyes and 0.7 (SD 0.23) in the left eyes (Fig 3).

### POAG study characteristics for African Americans

Of the 11,521 African Americans, 9,441 were over the age of 20 years and considered for downstream genetic association studies of POAG. Of these adults, 267 were identified as POAG cases (2.82%) and 4,813 as POAG controls. As might be expected for an age-related ocular disease, the median age of cases was older than controls (62 versus 54 years; Table 2). More than half of the cases and controls were female; and approximately half were hypertensive. On average, both cases and controls were obese (median body mass index > 30.0 kg/m^2^).

**Table 2:** Study population characteristics of POAG definite cases and controls among African Americans in EAGLE BioVU.

	Definite Cases > 20 yrs (SD)	Controls > 40 yrs (SD)
N	138	4813
Age at Diagnosis (years)	62.0 (12.0)	--
Age at Last Clinic (years)	--	54 (11.7)
Sex (% female)	63.7	60
Hypertensive (%)	55.1	46.6
BMI (kg/m2)	30.1 (6.7)	30.1 (8.0)
Diastolic (mm/Hg)	74.5 (8.1)	80 (33.6)
Systolic (mm/Hg)	134.5 (14.1)	124 (26.2)
Cholesterol (mg/dL)	183 (40.6)	161 (65.2)
HDL (mg/dL)	52.5 (25.0)	53 (38.6)
LDL (mg/dL)	103 (42.9)	99 (50.7)
Triglycerides (mg/dL)	125 (76.3)	98 (67.8)

Median values were calculated for the following: Age at POAG diagnosis was determined by the date of when POAG ICD-9 (365.11) was first mentioned in the records. Age at last clinic visit (LCV) was taken as the date of the last CPT mentioned in the records for controls. An individual was classified as hypertensive if he/she met one of three criteria: systolic blood pressure > 140 mm/Hg, diastolic blood pressure > 90 mm/Hg, or on hypertension medications all within a two year window of when they were diagnosed with POAG in cases and a two year window of their LCV date for controls. Blood pressure (systolic and diastolic), lipids (total cholesterol, high-density cholesterol, low-density cholesterol, and triglycerides), and body mass index (height and weight) were calculated from labs or measurements within two years of POAG diagnosis or LCV. Abbreviations: standard deviation (SD)

### Preliminary genetic association analysis results for *CDKN2B-AS1* region

In previous genetic association studies, the *CDKNSB-AS1*gene region has been associated with glaucoma clinical sub-types and quantitative glaucoma traits, such as CDR, POAG [30], and normal tension glaucoma in populations of European and Asian descent. *CDKNSB-AS1* was targeted for fine-mapping by the Illumina Metabochip, with 459 SNPs assayed in this region. In this preliminary analysis of African American adults from EAGLE BioVU after quality control, 135 definite POAG cases and 1,376 POAG controls (S2 Table) were tested for an association with 258 common variants (MAF > 5%) in the *CDKN2B-AS1* region using a logistic regression model adjusted for age and sex. Although some SNPs were nominally associated at p < 0.05 (S1 Fig), none passed a strict Bonferroni correction (p < 0.0001).

## Discussion

We developed a phenotype algorithm to extract POAG case and control status using a combination of structured and unstructured data available for research in the Vanderbilt de-identified EMR. The algorithm was implemented for 11,521 African Americans in EAGLE BioVU. Of those patients over the age of twenty (n = 9,441), we identified 267 cases of POAG and 4,813 controls without POAG by use of phenotype algorithms. Upon manual review of all POAG cases identified by the algorithm, 138 were classified as definite and 67 as potential, with the remaining 62 classified as false positives due to misclassification or missing data (S3 Table). Manual review of 300 controls confirmed control status among individuals identified by the algorithm. Evaluation of the algorithm revealed high performance for POAG control status (NPV = 98.3%) but low performance for POAG case status (PPV = 51.6% an accuracy = 76.3%).

Despite the low PPV of this POAG algorithm, we are encouraged that the algorithm has modest accuracy. This POAG algorithm was designed for an EMR environment lacking digital photographs for review in a research setting. We intentionally designed the algorithm to assist us in identifying all possible true POAG cases given that manual review of the EAGLE BioVU’s almost 10,000 African American adults would be prohibitively time and resource consuming. An alternative strategy to triaging records for manual review is the requirement of a single ICD-9 code for POAG. Based on these relaxed criteria, we identified 309 African Americans in EAGLE BioVU whose EMR contained an ICD-9 code for POAG. After review of these additional individuals for definite POAG case status, we determined that all measures of performance were lower (PPV = 47.2% and accuracy = 72.4%) compared with the algorithm developed here.

As evident by this study, broad phenotyping of a disease cohort is well within the capabilities of an EMR, even one with limited access to clinical data, through the use of simple text-mining techniques incorporating pattern matching and structured data from the EMR. The algorithm designed here for POAG is stringent. The PPV (51.6%) of this POAG algorithm for definite case status is well below the threshold of 95% adopted by consortia such as the electronic Medical Records & Genomics (eMERGE) network[31] for use in large-scale genetic association studies[3,29]. However, the addition of potential cases substantially increases the PPV to 76.7%. If we had designed the algorithm to merely detect an individual with a “general” glaucoma classification, the PPV increased to 87.2%. Furthermore, we have developed a highly discriminatory algorithm (NPV 98.3%) that can identify ocular controls. In the development of phenotype algorithms there is the constant tradeoff between overly strict or vague criteria resulting in loss of cases or misclassification of subjects, both of which will lead to a loss in statistical power in downstream genetic association studies.

Although most clinics within a health care organization that maintains an EMR adopt the digital system, occasionally a clinic may be excluded from converting to an all-digital interface or choose to opt out. The reasons for these exclusions may vary, but the consequences of missing data ultimately impact studies. Evaluation of the algorithm was limited by the lack of available funduscopic images (i.e., the “gold standard”) for validation, a limitation of this study. Missing data limited our ability to verify all POAG cases initially identified. Several cases identified here could only be classified as potential cases given the lack of sufficient clinical records to determine the sub-type of glaucoma. This limitation underscores the need for better implementation of EMRs across healthcare organizations for use in biomedical research.

Missing data can also introduce misclassification bias into studies. The control algorithm developed here was designed around the concept that an individual is free of POAG. However, without a complete medical work-up, there is the potential that a control is an undiagnosed case. Misclassification and potential ascertainment bias is also possible in case identification where cases are only those individuals who have been evaluated by a specialist and are therefore potentially extreme or overly symptomatic cases. A well-known barrier for individuals seeking medical attention is low socioeconomic status which disproportionally affects African Americans[32]. Additionally, at least one study suggests that the lifetime prevalence of undiagnosed glaucoma in African Americans is 50%[33]. This may in part explain the limited number of African American POAG cases (n = 267) representing only 2.82% of African Americans in EAGLE BioVU. Given the expected prevalence of POAG at 4–5% among African Americans[27,34], presumably the cases in EAGLE BioVU are only being diagnosed once vision loss becomes severe.

The age and gender compositions of the EAGLE BioVU definite POAG cases (Table 2) and controls differ when compared with other clinical and epidemiologic cohorts. More than half of EAGLE BioVU definite POAG cases (63.7%) and controls (60%) are female. In contrast, the International Consortium of African Ancestry Research in Glaucoma (ICAARE-Glaucoma; n = 2,150) study identified fewer female cases and controls[35]: African American cases (49.5%), African American controls (54.6%), Ghanaian cases (43.9%), and Ghanaian controls (57.3%). Also, the mean age at diagnosis for ICAARE-Glaucoma African American cases (57.0 years) is younger than EAGLE BioVU POAG cases (62.0 years) while the ICAARE-Glaucoma control group (59.4 years) is older compared with EAGLE BioVU POAG controls (54 years). These demographic differences are likely the result of differences in recruitment or ascertainment of African Americans within their respective communities, which can potentially introduce heterogeneity in downstream studies.

### Analysis of *CDKN2B-AS1* in African Americans

This study did not find a significant association in the *CDKN2B-AS1* region for POAG in African Americans. These results are not entirely surprising given the differences in susceptibility and linkage disequilibrium between European and African-descent populations (S2 and S3 Figs). Most of the significant associations between this gene and POAG were discovered in European and Japanese populations (Table 3) [30,36–38]. Of these SNPs, two were available on the Metabochip but were not found to be associated with POAG in this study. Our results are somewhat consistent with Liu et al [35] who investigated known European POAG risk loci in both an African American and Ghanaian population. Liu et al failed to replicate many of the European risk loci in their African populations, but they did identify one SNP (rs10120688) in *CDKN2B-AS1* significantly associated with POAG. This SNP is not available on the Metabochip and as such we could not replicate this association in our dataset. *CDKN2B-AS1* rs1333049 was available for analysis in this study, and interestingly, our results (OR = 0.72; p = 0.06) were similar to that reported Liu et al for the same SNP (OR = 0.89; p = 0.07) [35].

**Table 3:** Published index variants for the *CDKN2B-AS1 region* associated with POAG or POAG associated trait and availability of these variants on the Metabochip.

rs#	population	OR	p-value	Discovery study	Current Study	OR	p-value
rs7865618	Japanese	1.78	9x10-11	Nakano et al[36]	not available	-	-
rs1063192	Japanese	1.33	5x10-10	Osman et al[37]	-	0.92	0.75
rs2157719	European American	1.45	2x10-18	Wiggs et al[38]	-	0.97	0.92
rs4977756	European	1.50	4.7x10-9	Burdon et al[30]	not available	-	-
rs10120688	African American	1.21	0.002	Liu et al[35]	not available	-	-

Shown are significant index variants which are listed on the NHGRI GWAS catalog and within PubMed. Included is the availability of the index variants on the Metabochip and summary results for the current studies association analysis of African Americans with POAG in the *CDKN2B-AS1* region.

### Limitations and Conclusions

Our study has a number of limitations. The algorithm was developed at VUMC under the restrictions of a de-identified EMR, which limits access to certain data types, and the SD, which does not currently contain all pertinent medical records. As mentioned before the VEI does not utilize an electronic interface with structured data fields. Lack of these structured data fields hinders the extraction of ophthalmology exam results from the SD. Lack of exam results may prevent researchers from definitively ascertaining an individual’s ocular disease status. Due to these limitations it is unclear if our algorithm can be exported for use in the EMRs of other medical institutions. Additionally, one investigator performed the manual review preventing us from assessing intra- or inter-grader variability. This investigator was aware of the algorithm’s determination of case and control status, which may have introduced bias. However, we modeled our algorithm development process after the eMERGE Phenotype Working Group workflow[2,39]. In the eMERGE Network, algorithm development and assessment are an iterative process. That is, content experts design the initial algorithm and deploy it. After a round of manual reviews and performance calculations, the algorithm is altered, re-deployed, and re-evaluated. There is potential bias in reviewing and assessing the performance of the algorithm when adopting the eMERGE workflow.

Despite the limitations in portability and data access, we were able to define primary open-angle glaucoma from the VUMC’s SD. We have a diverse population connected to a depth of medical data, even if not all of it is easily searchable. Our study has made available more case counts for African Americans with ocular disease. And, BioVU continues to accrue samples as well as update the medical records associated with samples already collected; therefore, the accrual of additional cases is anticipated. The ability to extract ocular phenotypes from EMRs will provide researchers with previously unaccessed datasets to further advances in ocular genetic research and vision-loss prevention.

## Methods

### Ethics Statement

BioVU study subjects are not consented. DNA is collected from discarded blood samples remaining after routine clinical testing and is linked to de-identified medical records. According to the Vanderbilt Institutional Review Board (IRB) and the Federal Office of Human Research Protections provisions, the Vanderbilt protocol is considered nonhuman subjects research (The Code of Federal Regulations, 45 CFR 46.102 (f)). The IRB at Vanderbilt University approved this research.

### Population

The Epidemiologic Architecture for Genes Linked to Environment (EAGLE) study, as part of the Population Architecture using Genomics and Epidemiology (PAGE) I study[22], accesses clinical and epidemiological collections with racially/ethnically diverse populations. These collections are used to perform and generalize genetic association studies for common human diseases, including common ocular diseases such as POAG. As part of PAGE I, EAGLE genotyped all DNA samples in BioVU from non-European-descent individuals as of 2011 (EAGLE BioVU; n = 15,863; Table 2)[23]. All DNA samples were genotyped on the Illumina Metabochip by the Vanderbilt University Center for Human Genetics Research DNA Resources Core. The Metabochip is a custom array designed for replication and fine mapping of genome-wide association study (GWAS)-identified variants for metabolic and cardiovascular traits [40]. The data described here will be available through the database of Genotypes and Phenotypes (dbGaP).

### Development of Phenotype Algorithm

#### Initial screening criteria for study population

Individuals included for this POAG study were African American adults over the age of 20 years as of March 20, 2013. We excluded pediatric glaucoma cases because it is a separate condition caused by developmental issues prior to birth and/or by a very rare genetic mutation. The genetics of pediatric glaucoma are likely to be fundamentally different from the genetics of glaucoma in older adults, and this genetic heterogeneity would result in lower power to detect genetic associations for adult POAG. The final inclusion criteria required that an individual’s medical records include either one mention of a CPT code for ophthalmology or a CPT code for general clinic procedures (Fig 1).

#### Primary open-angle glaucoma cases

POAG cases were identified as individuals with at least one ICD-9 code for POAG (365.11). Cases were not excluded if they contained an additional ICD-9 code for another sub-type of glaucoma (list of glaucoma ICD-9 codes included in Fig 1). It is not uncommon for patients to experience different sub-types of glaucoma bilaterally or within the same eye which is also known as mixed- or combined- mechanism glaucoma. Also, over the course of a lifetime a patient may progress from one form to another such as closed-angle glaucoma to open-angle glaucoma.

#### Primary open-angle glaucoma controls

Controls were defined as individuals whose records were devoid of any glaucoma ICD-9 code. If the algorithm identified “glaucoma,” “glaucome,” “glocoma,” “gloucoma,” “gluacoma,” “glucoma,” or “glycoma” in a free text search of problems lists and clinical notes, the individual was excluded. We excluded individuals under the age of 40 years, as calculated from a given birth-date, to reduce contamination of controls with future cases.

#### Calculation of PPV, NPV, Sensitivity, Specificity, and Accuracy

The performance of the case and control algorithms were calculated as follows: PPV was calculated as the ratio of true positives (TP) over TP + false positive (FP) [PPV = TP/(TP + FP)]. A TP was an individual who was identified by the case algorithm as a case and was then confirmed by manual review to be a true case. A FP in turn was an individual identified as a case that was determined not to be a case during manual review. NPV is the ratio of true negative (TN) (i.e., a control who was confirmed as a control) over TN and FN [NPV = TN/(TN + FN)]. Lastly, Accuracy was calculated as the ratio of the sum of TP and TN over the sum of all positives and all negatives [Accuracy = (TP + TN)/(Positives (TP + FP) + Negatives (TN + FN))].

#### Extraction and calculation of individual demographic elements

Demographic data and laboratory measurements were extracted and calculated with an emphasis for use in future case/control studies. For cases, age at POAG diagnosis was determined by the date in the records for the first mention of a POAG ICD-9 (365.11). For controls, age at last clinic visit (LCV) was taken as the date of the last CPT mentioned in the records. An individual, regardless of case/control status, was classified as a hypertensive if he/she met one of three criteria: 1) systolic blood pressure > 140 mm/Hg, 2) diastolic blood pressure > 90 mm/Hg, or 3) mention of a hypertension medication within a two-year window of when an individual was diagnosed with POAG for cases or within a two-year window of his/her LCV date for controls. Median values were calculated for the following laboratory measurements within a two-year window of an individual’s POAG diagnosis or LCV for controls: blood pressure (systolic and diastolic), lipids (total cholesterol, high-density cholesterol, low-density cholesterol, and triglycerides), and body mass index (height and weight).

### Genetic association analyses

We tested for an association between POAG and 258 SNPs in the *CDKN2B-AS1* region of chromosome 14 in African Americans. Individuals included in this analysis were those identified as “definite” POAG cases regardless of age and POAG controls over the age of 60 years. Individuals passed quality control protocol for cryptic relatedness (based on identify-by-descent). Initially 459 SNPs were available for study. Each SNP considered for analysis passed quality control standards for HWE (p< 0.001), genotyping efficiency (>95%), and minor allele frequency (>5%). After quality control, 258 SNPs were available for analyses. Analyses were conducted using PLINKv1.07[41]. Each SNP was tested for an association using logistic regression assuming a log-additive genetic model adjusted by age and sex.

## Supporting Information

S1 Table List of glaucoma medications used in validation of primary open-angle glaucoma cases. Indications is a limited list of FDA approved uses as stated on the Drugs@FDA website as of October 4^th^, 2014: http://www.accessdata.fda.gov/scripts/cder/drugsatfda/.
(DOCX)

S2 Table Study population characteristics of POAG definite cases and controls over 60 years among African Americans in EAGLE BioVU. Median values were calculated for the following: Age at POAG diagnosis was determined by the date of when POAG ICD-9 (365.11) was first mentioned in the records. Age at last clinic visit (LCV) was taken as the date of the last CPT mentioned in the records for controls. An individual was classified as hypertensive if he/she met one of three criteria: systolic blood pressure > 140 mm/Hg, diastolic blood pressure > 90 mm/Hg, or on hypertension medications all within a two year window of when they were diagnosed with POAG in cases and a two year window of their LCV date for controls. Blood pressure (systolic and diastolic), lipids (total cholesterol, high-density cholesterol, low-density cholesterol, and triglycerides), and body mass index (height and weight) were calculated from labs or measurements within two years of POAG diagnosis or LCV. Abbreviations: standard deviation (SD)
(DOCX)

S3 Table Study population characteristics of total POAG cases and controls among African Americans in EAGLE BioVU. Median values were calculated for the following: Age was calculated from a given birth year. Age at POAG diagnosis was determined by the date of when POAG ICD-9 (365.11) was first mentioned in the records. Age at last clinic visit (LCV) was taken as the date of the last CPT mentioned in the records for controls. An individual was classified as hypertensive if he/she met one of three criteria: systolic blood pressure > 140 mm/Hg, diastolic blood pressure > 90 mm/Hg, or on hypertension medications all within a two year window of when they were diagnosed with POAG in cases and a two year window of their LCV date for controls. Blood pressure (systolic and diastolic), lipids (total cholesterol, high-density cholesterol, low-density cholesterol, and triglycerides), and body mass index (height and weight) were calculated from labs or measurements within two years of POAG diagnosis or LCV. Abbreviations: standard deviation (SD)
(DOCX)

S1 Fig Locus zoom plot of African American POAG association results for *CDKN2B-AS1*. Figure was generated using LocusZoom plot (http://csg.sph.umich.edu/locuszoom/) with no linkage disequilibrium calculations. Results are shown for the African American POAG association results in the *CDKN2B-AS1* region for the model adjusted by age and sex.
(TIF)

S2 Fig Locus Zoom plot of African American POAG association results for *CDKN2B-AS1* with LD calculations from 1000 Genomes YRI. Figure was generated using LocusZoom plot (http://csg.sph.umich.edu/locuszoom/) with linkage disequilibrium calculations from the hg18 1000 Genomes June 2010 YRI dataset. Results are shown for the African American POAG association results in the *CDKN2B-AS1* region for the model adjusted by age and sex.
(TIF)

S3 Fig Locus Zoom plot of African American POAG association results for *CDKN2B-AS1* with LD calculations from 1000 Genomes CEU. Figure was generated using LocusZoom plot (http://csg.sph.umich.edu/locuszoom/) with linkage disequilibrium calculations from the hg18 1000 Genomes June 2010 CEU dataset. Results are shown for the African American POAG association results in the *CDKN2B-AS1* region for the model adjusted by age and sex.
(TIF)
