# Supplementary material for: Extracting Primary Open-Angle Glaucoma from Electronic Medical Records for Genetic Association Studies
Source: PLoS One. 2015 Jun 10;10(6):e0127817. doi: 10.1371/journal.pone.0127817 (PMC4465698; doi:10.1371/journal.pone.0127817)
Supplement: S1 Table — Indications is a limited list of FDA approved uses as stated on the Drugs@FDA website as of October 4th, 2014: http://www.accessdata.fda.gov/scripts/cder/drugsatfda/. (DOCX) [file pone.0127817.s001.docx]

**Supplemental Table 1: List of glaucoma medications used in validation of primary open-angle glaucoma cases**

| **Drug name(s)** | **Generic drug name/active ingredient** | **Indications*** |
| --- | --- | --- |
| Diamox | Acetazolamide | Some types of glaucoma, epilepsy, and cardiac edema |
| Alphagan | Brimonidine | Prevention of elevated intraocular pressure (IOP)post operatively in individuals undergoing argon laser trabeculoplasty (ALT) |
| Iopidine | Apraclonidine | Prevention of elevated IOP post operatively in individuals undergoing ALT, argon laser iridotomy, or Nd:YAG posterior capsulotomy |
| Azopt | Brinzolamide | Treatment of elevated IOP for individuals with open angle glaucoma and ocular hypertension |
| Betoptic | Betaxolol | Treatment of elevated IOP for individuals with chronic open angle glaucoma and ocular hypertension |
| Cosopt | Dorzolamide and Timolol | Treatment for elevated IOP in individuals with open angle glaucoma or ocular hypertension |
| Trusopt | Dorzolamide | Treatment for elevated IOP in individuals with open angle glaucoma or ocular hypertension |
| Latanoprost/ Xalatan | Latanoprost | Treatment for elevated IOP in individuals with open angle glaucoma or ocular hypertension |
| Lumigan | Bimatoprost | Treatment for elevated IOP in individuals with open angle glaucoma or ocular hypertension |
| Timoptic(-xe)/ Betimol/ Istalol/ Blocadren | Timolol maleate | Treatment for elevated IOP in individuals with open angle glaucoma or ocular hypertension |
| Travatan | Travoprost | Treatment for elevated IOP in individuals with open angle glaucoma or ocular hypertension |

Indications is a limited list of FDA approved uses as stated on the Drugs@FDA website as of October 4^th^, 2014: http://www.accessdata.fda.gov/scripts/cder/drugsatfda/
