# Supplementary material for: Extracting Primary Open-Angle Glaucoma from Electronic Medical Records for Genetic Association Studies
Source: PLoS One. 2015 Jun 10;10(6):e0127817. doi: 10.1371/journal.pone.0127817 (PMC4465698; doi:10.1371/journal.pone.0127817)
Supplement: S2 Table — Median values were calculated for the following: Age at POAG diagnosis was determined by the date of when POAG ICD-9 (365.11) was first mentioned in the records. Age at last clinic visit (LCV) was taken as the date of the last CPT mentioned in the records for controls. An individual was classified as hypertensive if he/she met one of three criteria: systolic blood pressure > 140 mm/Hg, diastolic blood pressure > 90 mm/Hg, or on hypertension medications all within a two year window of when they were diagnosed with POAG in cases and a two year window of their LCV date for controls. Blood pressure (systolic and diastolic), lipids (total cholesterol, high-density cholesterol, low-density cholesterol, and triglycerides), and body mass index (height and weight) were calculated from labs or measurements within two years of POAG diagnosis or LCV. Abbreviations: standard deviation (SD) (DOCX) [file pone.0127817.s002.docx]

**Supplemental Table 2: Study population characteristics of POAG definite cases and controls over 60 years among African Americans in EAGLE BioVU**

|  | Definite Cases > 20 yrs (SD) | Controls >60 yrs (SD) |
| --- | --- | --- |
| N | 138 | 1376 |
| Age at Diagnosis (years) | 62.0 (12.0) | -- |
| Age at Last Clinic (years) | -- | 67.3 (7.8) |
| Sex (% female) | 63.7 | 56.5 |
| Hypertensive (%) | 55.1 | 52.5 |
| BMI (kg/m^2^) | 30.1 (6.7) | 28.8 (7.35) |
| Diastolic (mm/Hg) | 74.5 (8.1) | 76.0 (8.8) |
| Systolic (mm/Hg) | 134.5 (14.1) | 135 (14.6) |
| Cholesterol (mg/dL) | 183 (40.6) | 169 (46.7) |
| HDL (mg/dL) | 52.5 (25.0) | 49 (17.8) |
| LDL (mg/dL) | 103 (42.9) | 93 (37.4) |
| Triglycerides (mg/dL) | 125 (76.3) | 97 (68.1) |

Median values were calculated for the following: Age at POAG diagnosis was determined by the date of when POAG ICD-9 (365.11) was first mentioned in the records. Age at last clinic visit (LCV) was taken as the date of the last CPT mentioned in the records for controls. An individual was classified as hypertensive if he/she met one of three criteria: systolic blood pressure > 140 mm/Hg, diastolic blood pressure > 90 mm/Hg, or on hypertension medications all within a two year window of when they were diagnosed with POAG in cases and a two year window of their LCV date for controls. Blood pressure (systolic and diastolic), lipids (total cholesterol, high-density cholesterol, low-density cholesterol, and triglycerides), and body mass index (height and weight) were calculated from labs or measurements within two years of POAG diagnosis or LCV. Abbreviations: standard deviation (SD)
