# Supplementary figures and images for: Extracting Primary Open-Angle Glaucoma from Electronic Medical Records for Genetic Association Studies
Source: PLoS One. 2015 Jun 10;10(6):e0127817. doi: 10.1371/journal.pone.0127817 (PMC4465698; doi:10.1371/journal.pone.0127817)

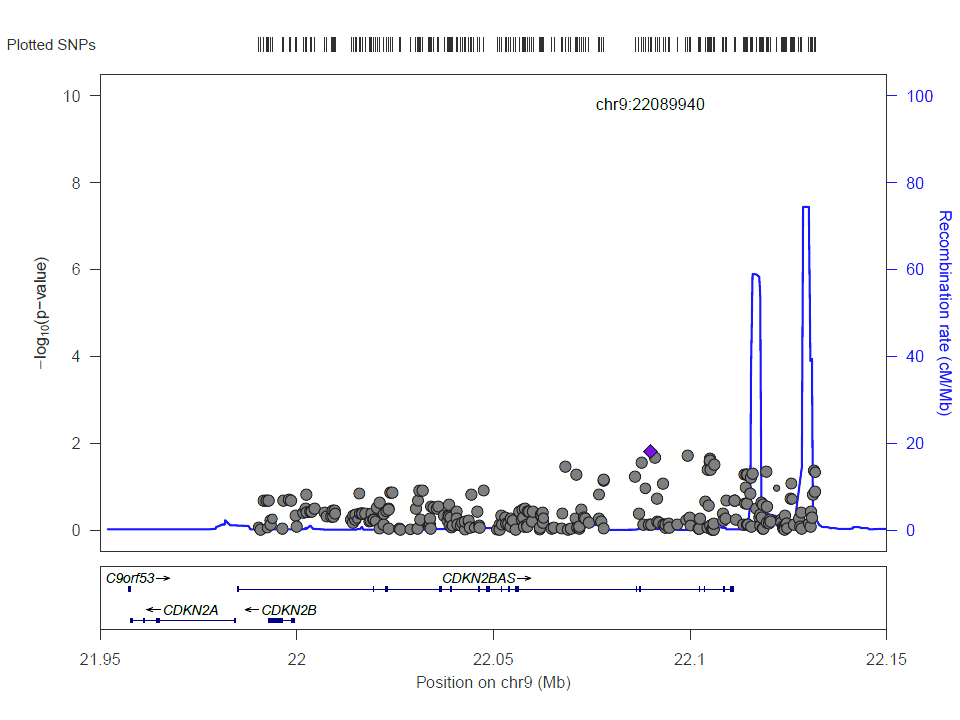

Supplement: S1 Fig — Figure was generated using LocusZoom plot (http://csg.sph.umich.edu/locuszoom/) with no linkage disequilibrium calculations. Results are shown for the African American POAG association results in the CDKN2B-AS1 region for the model adjusted by age and sex. (TIF) [file pone.0127817.s004.tif]

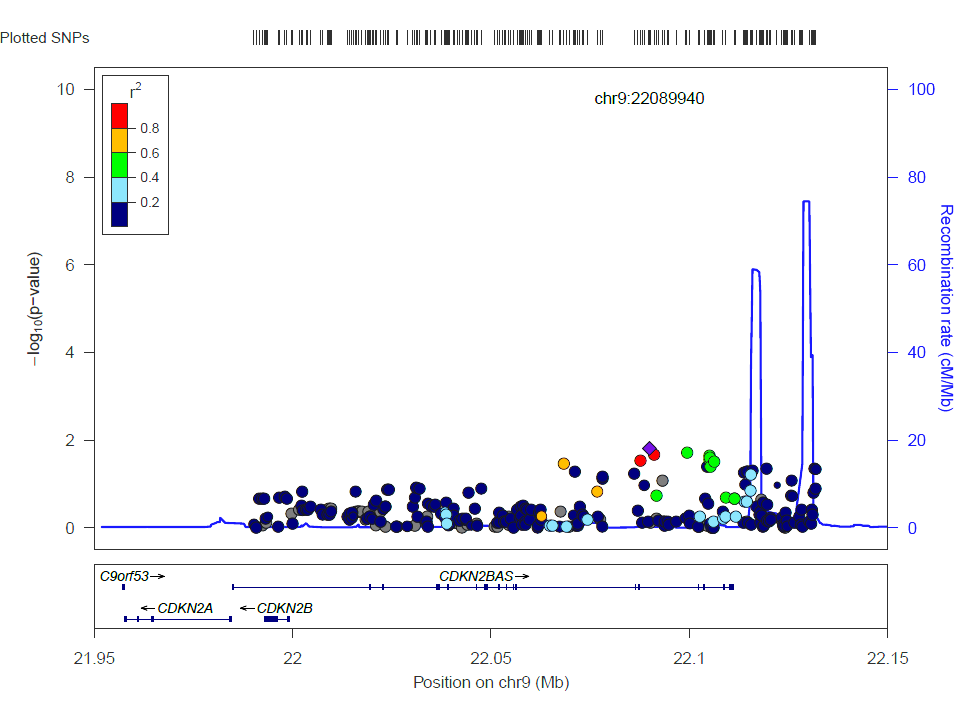

Supplement: S2 Fig — Figure was generated using LocusZoom plot (http://csg.sph.umich.edu/locuszoom/) with linkage disequilibrium calculations from the hg18 1000 Genomes June 2010 YRI dataset. Results are shown for the African American POAG association results in the CDKN2B-AS1 region for the model adjusted by age and sex. (TIF) [file pone.0127817.s005.tif]

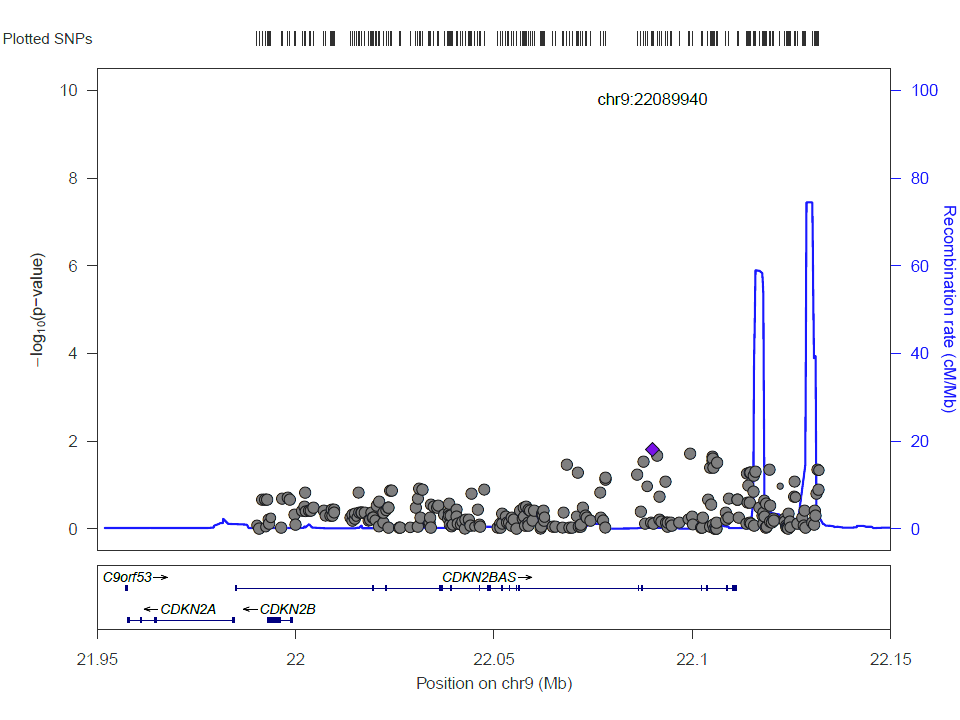

Supplement: S3 Fig — Figure was generated using LocusZoom plot (http://csg.sph.umich.edu/locuszoom/) with linkage disequilibrium calculations from the hg18 1000 Genomes June 2010 CEU dataset. Results are shown for the African American POAG association results in the CDKN2B-AS1 region for the model adjusted by age and sex. (TIF) [file pone.0127817.s006.tif]
